# Lablab (*Lablab purpureus* L.) genotypes and field margin vegetation influence bean aphids and their natural enemies

**DOI:** 10.3389/finsc.2024.1328235

**Published:** 2024-06-28

**Authors:** Joseph M. Karimi, Jane G. Nyaanga, Richard M.S. Mulwa, Joshua O. Ogendo, Philip K. Bett, Erick K. Cheruiyot, Sarah E.J. Arnold, Steven R. Belmain, Philip C. Stevenson

**Affiliations:** ^1^ Department of Crops, Horticulture and Soils, Egerton University, Egerton, Kenya; ^2^ Department of Biological Sciences, Egerton University, Egerton, Kenya; ^3^ Department of Sustainable Agriculture, Nelson Mandela African Institution of Science and Technology, Arusha, Tanzania; ^4^ NIAB, East Malling, Kent, United Kingdom; ^5^ Natural Resources Institute, University of Greenwich, Chatham Maritime, United Kingdom; ^6^ Trait Diversity and Function, Royal Botanic Gardens Kew, Richmond, United Kingdom

**Keywords:** bean aphids, lablab, field margin vegetation, natural enemies, natural pest regulation

## Abstract

Lablab (*Lablab purpureus* L.) is an important food and livestock feed legume that can also enhance soil fertility. However, its production is limited by insect pests, notably the black bean aphid (*Aphis fabae*). The present field study was conducted to determine the difference in the contribution of lablab genotypes and natural field margin vegetation (FMV) to the abundance and diversity of natural enemies and the damage, incidence, and abundance of bean aphids. Eighteen lablab genotypes were planted in the presence or absence of FMV in a randomized complete block design experiment replicated four times. Data on aphid abundance, incidence, and severity of damage were collected at four growth stages of the crop. Lablab genotypes significantly influenced aphid incidence, suggesting some level of tolerance to aphid colonization. Findings showed that lablab genotypes were a significant influence on natural enemy species richness with no statistical difference for abundance and natural enemy species diversity. However, the genotypes did not vary significantly in their influence on the number of aphid natural enemies. FMV was associated with low bean aphid damage. Overall, the presence or absence of FMV did not influence the number of natural enemies caught on the crop. This concurs with recent work that shows a similar number of natural enemies with field margin plants but may reflect the reduced number of pest insects. Cropping seasons influenced aphid abundance and damage severity, with the populations developing at the early stages of lablab development and decreasing as the crop advanced. This pattern was similar both in the presence or absence of FMV. The findings of this study highlight the important contribution of crop genotype together with the presence of field margin species in the regulation of aphids and their natural enemies in lablab.

## Introduction

Lablab (*Lablab purpureus* L.) also known as dolichos and as *njahi* in Kenya is a multipurpose legume grown as a pulse, vegetable, and livestock feed. It is a drought-tolerant and nutritious legume with great potential to mitigate the impact of climate change on food and nutritional security; moreover, it plays a major economic role for many households (1, 2). In Africa and Asia, the immature pods and young leaves are cooked as vegetables and the mature dry beans as pulses (3). Lablab is especially rich in proteins (4) and nutritionally superior compared to other legumes such as soybean (*Glycine max*) and common bean (*Phaseolus vulgaris*) (5, 6). The protein content of the seeds has been reported to be up to 25% (7). Lablab is also a good source of dietary fiber, carbohydrates, calcium, phosphorus, and iron (8).

Lablab is also a fast-growing crop that can be used as a cover crop to protect the soil from erosion or plowed in as green manure to improve soil fertility (9). Despite its importance and benefits, lablab remains neglected and arguably underutilized in terms of area under cultivation and scientific efforts toward genetic enhancement (10).

The yield of lablab in Kenya ranges from 800 to 900 kg ha^−1^ against a potential of 3,000 kg ha^−1^ (11, 12). Low yields make the crop less preferred by farmers compared to other legumes despite its drought tolerance. The low yields have been partially attributed to factors such as low-yielding cultivars, the use of unimproved germplasm, and high susceptibility to insect pests, like bean aphids (*Aphis fabae* and *Aphis craccivora*) (13). The nymphal and adult stages of aphids feed on lablab by sucking sap from the leaves, petiole, stems, inflorescence, and tender pods of the crop (14). The affected leaves often wilt and curl causing the crop to remain stunted, and in severe cases, the crop withers or dies. The incidence, severity, and extent of loss due to this pest differ across seasons, locations, and cropping systems (15).

Yield losses caused by insect pests are widespread among smallholder farmers leading to the widespread use of synthetic pesticides (16). Losses to aphids range from 40% to 90% (3). The overreliance on synthetic pesticides poses serious risks to the environment and human health (17). Host plant resistance and biological control are two methods that have attracted attention as alternatives to synthetic pesticides. Despite significant efforts in recent years to understand the mechanisms of insect resistance in grain legumes, as well as screening and selecting aphid-tolerant and aphid-resistant genotypes, there has been limited success with lablab (18) as it is considered a neglected orphan crop. While lablab is recognized for its genetic diversity, there are similar impediments to the widespread adoption of insect-resistant cultivars in other legumes (3, 19). Inadequate seed production and distribution efforts, as well as the lack of investment in research and development, are among the obstacles (20).

The implementation of cultural approaches that combine good agricultural practices (GAPs), such as deliberate modification of the crop and its environment as well as intercropping, can boost natural enemy activity (21). The use of natural enemies for biological control is long-term and cost-effective compared to conventional methods in the long run (22). As a result of the loss of natural habitats surrounding croplands, natural enemies are forced to disperse from decreasing and more distant non-crop reservoirs. Field margin vegetation (FMV) creates a niche for supplementary food, population expansion, and refuge of natural enemies which are key components of natural pest regulation (23). The loss of natural habitats surrounding croplands could lead to natural enemy population reduction to distant non-crop reservoirs, while the use of pesticides reduces their abundance further. Insect-resistant genotypes offer an additional pest management alternative to pesticides. However, in lablab, research on insect resistance is limited to storage pests (24). Combining both natural enemies and resistance crop varieties could lead to a more effective pest control approach, but an evaluation of their individual and combined effects on insect pests and natural enemies, as well as their overall impact on crop yield, is required. There is also increasing evidence that shows that the qualities of pest-infested plants influence the behavior of natural enemies. Plant traits could therefore influence both the numerical and functional responses of natural enemies (25–27). This study investigated the potential contribution of genotype and natural field margin vegetation on crop damage and abundance of bean aphids and their natural enemies in lablab.

## Materials and methods

### Experimental site

The study was conducted at the agronomy research field (0°22′S, 35°55′E) of Egerton University during the 2019 and 2020 cropping seasons. The field lies at an altitude of 2,238 m above sea level (m.a.s.l) classified as agroecological zone III under lower highland 3 (28). The location receives an annual precipitation of approximately 1,200 mm with a bimodal distribution. The average maximum temperature of this location is 22°C with a minimum of 17°C. The weather data were captured during the study periods (**Table 1**). The soils are predominantly mollic Andosols, which are well-drained dark reddish clays.

**Table 1:** Mean monthly rainfall, relative humidity, and temperature data recorded during the study period: June to December 2019 and May to November 2020.

	2019				2020			
	Temp (%)		Rain (mm)	RH (%)	Temp (%)		Rain (mm)	RH (%)
	Max.	Min.			Max.	Min.		
**May**	24.7	15.5	60.4	60	23.7	14.4	91.6	65
**June**	22.4	14.6	232.4	79	22.7	13.2	93.5	74
**July**	22.2	13.5	146.7	70	21.4	16.3	113.9	71
**August**	22.5	13.1	76.4	69	21.3	18.2	119.2	76
**September**	24.7	13.3	89.7	65	22.6	15.1	96.4	73
**October**	22.9	14.1	161.6	70	24.7	17.4	85.2	64
**November**	22.8	14.4	114.8	70	23.9	15.1	49.7	61
**December**	21.1	14.3	223.6	75	24.3	16.8	51.2	63

Source: Egerton University Engineering Meteorological Station, 2019 and 2020.

Temp, temperature (°C); Rain, rainfall (mm); RH, relative humidity.

### Germplasm used

The following 18 genotypes were sourced from different counties in Kenya and were named as follows: Machakos I, EUD1; Machakos II, EUD2; Machakos III, EUD3; Machakos Kiboko, EUD4; Eldo KTL-Black I, EUD5; CIAT 22759, EUD6; Echo Cream, EUD7; Brown Rongai, EUD8; Black Rongai, EUD9; CPI 81364, EUD10; DL1002, EUD11; Kikuyu Mkt, EUD12; Tx-24, EUD13; Q6880B, EUD14; Eldo KT Cream, EUD15; Kikuyu X-Meru, EUD16; Eldo KT-Black II, EUD17; and HA-4, EUD18.

### Experimental procedure

The different genotypes were planted in experimental units measuring 3.0 m × 2.5 m in a randomized complete block design (RCBD) factorial arrangement and replicated four times. Each genotype was planted in the presence or absence of FMV. To enhance the field margin vegetation, seeds from four plant species (*Bidens pilosa*, *Tagetes minuta*, *Ageratum conyzoides*, and *Galinsoga parviflora*), which were selected based on their relative abundance in the ecosystem and their positive influence on natural pest regulation from previous studies (21, 29, 30), were mixed in equal proportions and sown in the field margins. The FMV seed mixtures were sown 2 weeks before the lablab crop was planted to ensure they were flowering at the best time to offer benefit to natural enemies through nectar provision. The margin species were planted 0.5 m from the outer row of the lablab crop with a width of 0.5 m. The different lablab genotypes were planted at an inter- and intra-row spacing of 60 cm and 30 cm, respectively. At the time of planting, each plot received an equivalent rate of 13.8 kg N ha^−1^ and 13.8 kg P_2_O_5_ ha^−1^ with NPK (23:23:0) fertilizer as a source. Arthropods are known to be highly mobile (31); therefore, to minimize the movement of insects within the experimental plots, all surrounding vegetation was cleared throughout the growing season except for the FMV.

Weed control within the experimental plots was restricted to manual weeding at 21 and 49 days after planting (DAP). No insecticide was applied to allow for the natural population of bean aphids and their associated natural enemies.

### Data collection

#### Abundance, severity, and incidence of bean aphid

Data on aphid abundance, severity, and incidence were collected from 10 randomly selected plants within the three inner rows in each plot at the seedling, early vegetative, late vegetative, flowering, and podding growth stages. Due to the rapid reproductive rate of aphids, aphid abundance was scored using a 5-point scale, where 0 = no aphids, 1 = a few scattered aphids, 2 = a few small colonies, 3 = several small colonies, 4 = large isolated colonies, and 5 = large continuous colonies as used in the previous studies of Lumbierres et al. (32), Mkenda et al. (33), Ochieng et al. (34), and Ndakidemi et al. (35). The damage severity in each experimental plot was assessed by scoring the extent of damage using the following grades: 0 = no damage, 1 = showing damage up to 25%, 2 = damage from 26% to 50%, 3 = damage from 51% to 75%, and 4 = damage more than 75% (33), where 0 = no aphids, 1 = 0–50, 2 = 50–150, 3 = 151–650, 4 = 650–2,500, and 5 > 2,500/plant after Lumbierres et al. (32). The percent incidence of aphids was determined by visually examining and counting the number of plants infested with bean aphid and then expressed as a percentage of the total number of plants examined. Bean aphid incidence was assessed on a 0–1 scale (where 0 = clean plant with no signs of aphid infestation and 1 = plant with signs of aphid infestation) and expressed as percentage incidence using the following formula:


$$\text{Aphid incidence}\left(\%\right)=\frac{\text{Number of infested plants}}{\text{Total number of plants observed}}\times 100$$

#### Abundance of natural enemies of black bean aphids

Sampling of natural enemies was done using yellow pan traps comprising a 20-cm-diameter plastic dish filled with water and three droplets of detergent to break the surface tension and prevent trapped insects from crawling or flying away. The pan traps were placed at the center of the plot (middle of the inner two rows) and at the field margin. The traps were collected after 48 h and the invertebrate taxa trapped were transferred to 50 ml falcon tubes with 75% ethanol. The use of pan traps to measure populations of natural enemies is well established (36, 37). Pan traps can be deployed easily in the crop, catching insects throughout the deployment period, whereas other approaches such as sweep netting may be biased toward daytime-active insects and may miss nocturnal arthropods and tiny insects like parasitoid wasps. Invertebrate taxa samples were sorted, and key selected families of natural enemies associated with aphids (parasitic wasps, tachinid flies, ladybird beetles) were identified up to the family level using a Leica Zoom 2000 microscope (Leica Microsystems Inc., Buffalo, NY, USA 185 14240–0123). Identification was done up to the family level using Simon and Schuster’s identification key (38), and then the natural enemies were counted by taxa and recorded.

#### Growth, yield, and yield components of lablab genotypes

Morpho-agronomic trait evaluation of the genotypes was carried out. Qualitative and quantitative traits, mainly yield-related components, were measured at the seedling, vegetative, and reproductive growth stages on all the genotypes. The qualitative traits that were measured as per the descriptor list of the genus *Lablab* included the seed and flower color and leaf hairiness (pubescence).

The quantitative traits that were taken included stand count, days to 50% flowering, plant height, pod length, number of pods per plant, number of seeds per pod, peduncle length, aboveground biomass, and grain yield for each genotype. To remove the border effect, five plants were randomly selected within the three inner rows in each plot for data collection except for stand count that was determined by counting all the plants per plot 3 weeks after planting. Days to 50% flowering was determined by counting the days taken by each genotype from emergence to the stage when 50% of the plants flowered. The height of the plant was measured from the ground up to the main stem’s tip. Days to maturity was from emergence to the stage when 50% of the plants have mature pods. Destructive sampling was done at the pod set and data were used to determine the aboveground biomass for each genotype. Plant stems were cut above ground and immediately weighed to determine fresh weight. Samples were taken to the laboratory and oven-dried at 65°C for 48 h and then weighed. The biomass was expressed on a hectare basis using the following formula:


$$\text{Biomass }\left(\text{kg}/\text{ha}\right)=\text{Biomass }\left({\text{kg m}}^{-2}\right)\times 10,000.$$

Plant samples were dried in an oven at 65°C to attain constant weight. Ten pods per plant were counted in each genotype and subsequently opened to count the number of seeds in each pod. For grain yield, pods were harvested separately within the sampling area for each genotype. The pods were sun-dried and threshed on the third day, and their moisture content was determined using a digital moisture meter. After reaching a moisture content of 13%, the grains of each genotype were weighed separately on a portable digital scale to determine their relative weights. The grain collected for each genotype in the sampling region was converted into tons ha^−1^ using the formula below:


$$\text{Grain Yield }(\text{kg}/\text{ha})=\frac{\text{PW }(\text{kg})\;\times \;(100\;-\;{\text{AMC})\;\times \;10,000\text{ m}}^{2}/\text{ha}}{{\text{HA }(\text{m}}^{2})\;\times \;(100\;-\;\text{SMC})}$$

Where PW = plot weight (kg), AMC = actual moisture content at harvest, HA = harvest area (in m^2^), and SMC = safe storage moisture content (13.0%).

### Data analysis

Data on yield, biomass measures per hectare, and the natural enemy counts were subjected to log transformation as they were positively skewed. Additionally, data on yield and related measures (biomass/ha, pod count, pod length, peduncle length, days till 50% flowering) were analyzed using linear mixed models in R packages. The respective aphid infestation measures (aphid abundance, severity, and incidence) and natural enemy abundance (for Tachinidae, Syrphidae, Coccinellidae, and wasps) were also analyzed using linear mixed models in R packages lme4 (39) and lmerTest (40). Approximate *p*-values were generated from lmerTest using a type III analysis of variance (ANOVA) applying Kenward–Roger’s method (41).

The model design included genotype, margin, and their interaction as fixed factors, with replicate (for yield/pest populations) and season as random factors, and this was used to correct the considerable variability between years and replicates noted during initial data exploration.

Finally, to explore the relationship between natural enemies, pest populations, and yield, a correlation matrix was built and reshaped (using reshape2; 42) and then formed into a heatmap signifying the intensity of correlations visually. Data were visualized using ggplot2 (43) (**Figure 5**).

### Natural enemy abundance and diversity

The Shannon–Weaver index was used to evaluate the species diversity of natural enemies of bean aphids in each lablab genotype as well as within field margin vegetation. The number of natural enemies recorded on each lablab genotype at each growth stage in each of the two field margin vegetation types was utilized as the unit of measurement in this study. To estimate the diversity of insect families, the Shannon–Weaver index (*H*) formula below was used.


$$H=-\Sigma p_{i}lnp_{i}$$

*H* is the Shannon’s diversity index, *p_i_* is the proportion of individuals found in the *i*th species, and ln is the natural log of individuals found in the *i*th species.

## Results

### Bean aphid abundance, incidence, and severity of damage

**Figure 1:**
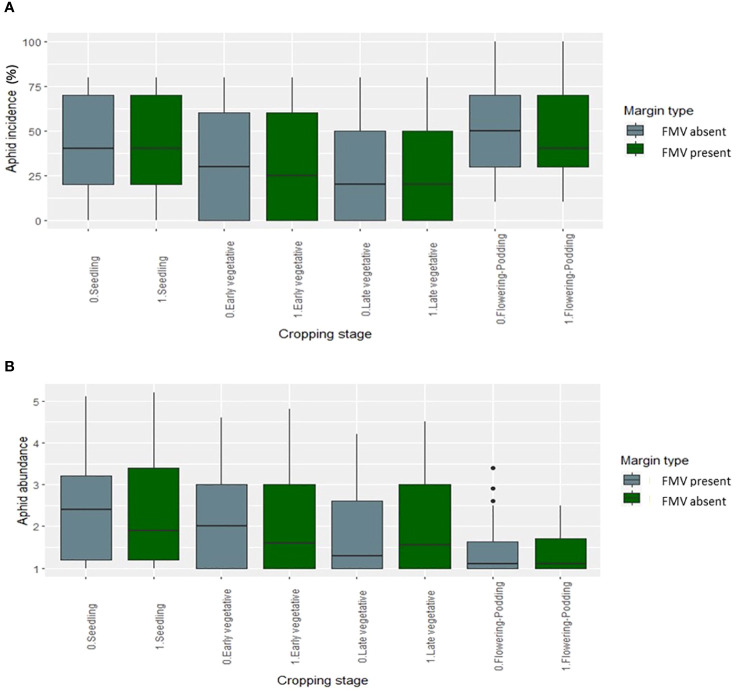
Aphid **(A)** incidence (%) and **(B)** mean abundance score (0–5) over the seasons in the presence and absence of FMV.

**Figure 2:**
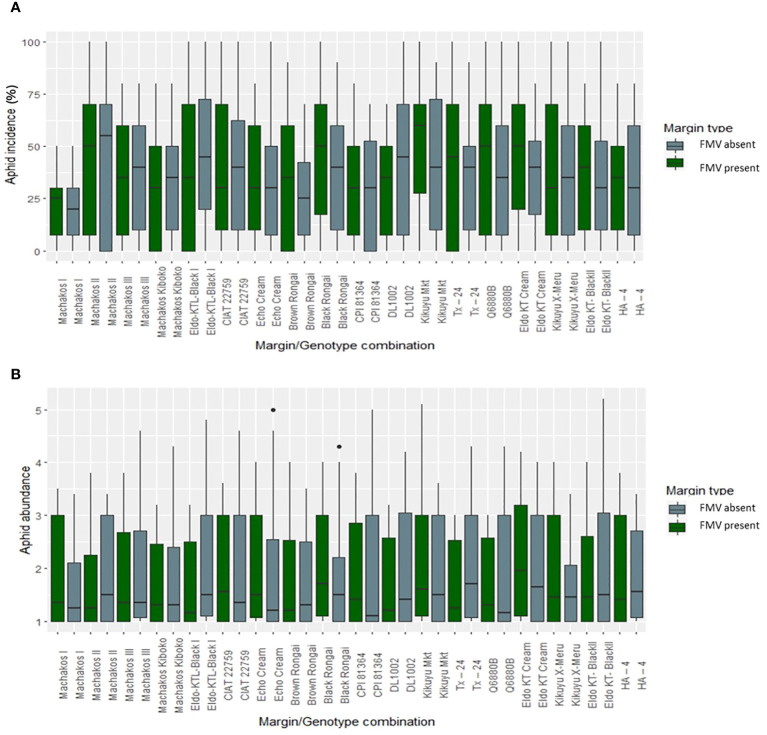
Aphid **(A)** incidence (%) and **(B)** mean abundance score (0–5) according to genotype × FMV interaction.

FMV had no significant effect on aphid abundance, incidence, and severity in the 2019 and 2020 cropping seasons (**Figure 1**). Overall, aphid abundance varied over the two cropping seasons, generally peaking in the early growth stages and decreasing later (**Figure 1A**). This pattern was similar regardless of the presence or absence of the field margin. The lablab genotypes showed differences in bean aphid incidence and abundance (**Figures 2A, B**); aphid incidence was significantly affected by lablab genotypes (*F*_1, 1,141_ = 5.7960, *p* = 0.01622). Machakos II recorded a high bean aphid incidence, while Brown Rongai recorded a low bean aphid incidence (**Figure 2A**). Eldo KT Cream recorded a high bean aphid abundance, while Brown Rongai recorded a low bean aphid abundance (**Figure 2B**).

### Natural enemy abundance and species diversity

**Figure 3:**
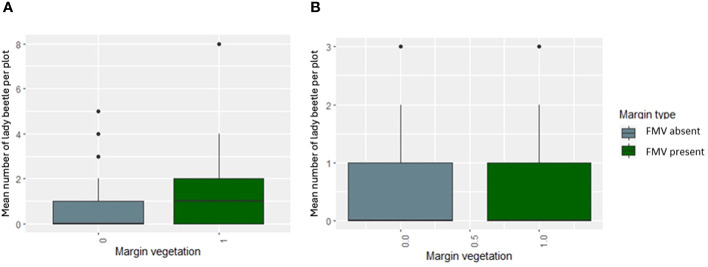
The mean number of lady beetle per plot in **(A)** 2019 and **(B)** 2020 in the presence or absence of FMV.

**Figure 4:**
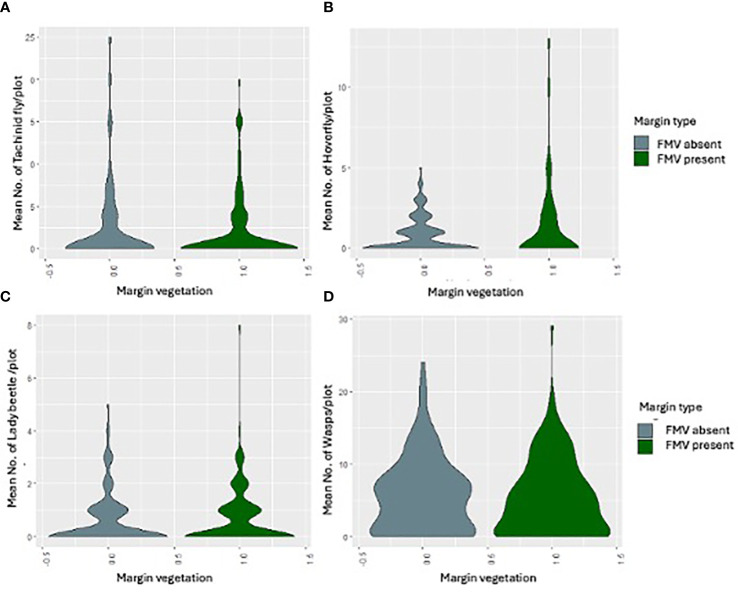
The mean number of **(A)** tachinid fly, **(B)** hoverfly, **(C)** lady beetle, and **(D)** wasps in the presence or absence of FMV across the two seasons.

The natural enemies were Tachinidae (*F*_1, 283_ = 0.9872, *p* = 0.3213), hoverfly (*F*_1, 283_ = 0.2564, *p* = 0.6130), lady beetle (*F*_1, 283_ = 2.5194, *p* = 0.1136), and wasps (*F*_1, 283_ = 0.1187, *p* = 0.7307) (**Figure 1**), and their numbers were not influenced significantly by the lablab genotype (**Table 2**). Lablab genotypes did not vary in their capacity to support natural enemies (**Table 2**). Equally, the presence or absence of field margin vegetation did not influence the number of natural enemies caught on the crop. That said, in 2019, there was a weak propensity for field margins to better support Coccinellidae (lady beetle) although this was not the case in the second cropping season (**Figure 3**). The abundance of Tachinidae, Syrphidae, Coccinellidae, and wasps was significantly high on plots with FMV across both seasons as compared to plots with no field margins. During the first cropping season, there was no significant difference in the insect invertebrate’s species diversity in the presence and absence of field margin vegetation. In the second cropping season, a significantly lower insect species diversity was recorded both in the presence and absence of field margin vegetation. In plots with field margins, a higher abundance of natural enemies was recorded in the center of the crop during the 2019 and 2020 cropping seasons as compared to the abundance in the margin vegetation (**Table 3**). Among the natural enemy groups collected were mainly predators and parasitoids which included tachinid flies (Diptera: Tachinidae), hoverflies (Diptera: Syrphidae), parasitic wasps (Ichneumonidae and Braconidae), and ladybird (Coleoptera: Coccinellidae) (**Figure 4**). A correlation analysis revealed that there was no significant predictive relationship between natural enemies and aphid populations (**Figure 4**).

**Table 2:** *p*-values from the linear mixed model (LMM) examining the effect of margin, genotype, and margin × genotype interaction on natural enemies.

	Margin	Genotype	Margin × genotype
**Tachinidae**	0.3213	0.1034	0.3197
**Syrphidae**	0.6130	0.6596	0.2335
**Coccinellidae**	0.1136	0.6415	0.1864
**Hymenoptera**	0.7307	0.3984	0.8819

**Table 3:** Effect of FMV and trapping area on the richness, abundance, and diversity of natural enemies of bean aphids during the 2019 and 2020 cropping seasons.

Field margin vegetation	2019 cropping season			2020 cropping season		
	Richness	Abundance	Diversity	Richness	Abundance	Diversity
Present	4.18^a^	12.29^b^	0.64^a^	3.36^b^	8.71^b^	0.26^b^
Absent	4.13^a^	17.35^a^	0.64^a^	4.77^a^	13.65^a^	0.32^a^
**Msd**	**0.32**	**2.50**	**0.03**	**0.46**	**1.88**	**0.02**
Trapping area						
Crop center	4.33^a^	16.72^a^	0.65^a^	4.69^a^	12.81^a^	0.31^a^
Margin vegetation	3.83^b^	4.49^b^	0.62^a^	2.11^b^	5.43^b^	0.23^b^
**Msd**	**0.32**	**2.50**	**0.03**	**0.46**	**1.88**	**0.02**

Means in a column followed by the same letters are not significantly different using Tukey’s HSD test at p ≤0.05.

### Morphological traits, growth, and yield of lablab genotypes

The presence or absence of FMV had no effect on yield and yield components of lablab genotypes, and we found no interaction between FMV and genotype (**Table 4**). Lablab genotypes differed in some aspects of plant development, specifically the number of pods (per plant), the number of peduncles, and peduncle length. The biomass of genotypes was significant at a significant level of *p ≤*0.05 (**Table 4**). Morphological trait characterization was based on visual observations of growth habits and structure of the plants. The variation of major qualitative and quantitative characteristics was observed across the genotypes. Maturity duration, the presence or absence of pubescence, and flower color varied across the genotypes (**Table 5**) to a greater extent. In crop establishment, the majority of genotypes had a higher number of stand count with Kikuyu Market and Kikuyu Meru having the highest population, while Echo Cream had the lowest population of stand count. A possible interaction could have explained the better performance of some genotypes in terms of yield and biomass, with genotypes that recorded low aphid populations (and possibly high numbers of natural enemies) producing more yields/biomass, but this was not the case. A correlation analysis revealed that there was no significant predictive relationship between yield and aphid populations (**Figure 4**).

**Table 4:** *p*-values from the linear mixed model (LMM) Kenward–Roger approximations for each combination of output variable.

	Genotype	Margin (presence/absence)	Genotype × margin
**Yield/ha**	0.3751	0.4364	0.7384
**Biomass/ha**	0.09942*	0.13580	0.45150
**Pod count**	0.04911**	0.12155	0.21598
**Pod length**	0.8329	0.9990	0.6867
**Peduncle count**	0.02585**	0.86890	0.44491
**Peduncle length**	0.01469**	0.94196	0.97666
**Days to 50% flowering**	0.1755	0.7365	0.6028
**Plant height**	0.1702	0.9841	0.9040

*, **, and ***: significance at p ≤ 0.05, p ≤ 0.01, and p ≤ 0.001, respectively.

**Table 5:** Agromorphological characteristics of different lablab genotypes.

Genotype	Maturity	Seed color	Flower color	Qualities	Other properties
Machakos I	Mid	Black	Purple	Pubescent	Dual purpose
Machakos II	Mid	Brown-spotted	Purple		Dual purpose
Machakos III	Mid	Brown	White		Grain variety
Machakos Kiboko	Late	Black	Purple		Grain variety
Eldo KTL-Black I	Late	Black	Purple	Pubescent	Grain variety
CIAT 22759	Late	Black	Purple		Forage variety
Echo Cream	Late	Cream	White		Forage variety
Brown Rongai	Mid	Brown	Purple	Pubescent	Forage variety
Black Rongai	Late	Black	Purple		Dual purpose
CPI 81364	Late	Brown	White	Pubescent	Grain variety
DL1002	Late	Black	Purple		Grain variety
Kikuyu Mkt	Late	Black	Purple		Grain variety
Tx-24	Late	Cream	Purple		Forage variety
Q6880B	Late	Black	Purple		Dual purpose
Eldo KT Cream	Late	Brown	White		Grain variety
Kikuyu X-Meru	Late	Black	Purple		Grain variety
Eldo KT-Black II	Mid	Black	Purple		Grain variety
HA-4	Early	Cream	White	Pubescent	Dual purpose

Early = 72–85 days; Mid = 85–99 days; Late = above 100 days.

**Figure 5:**
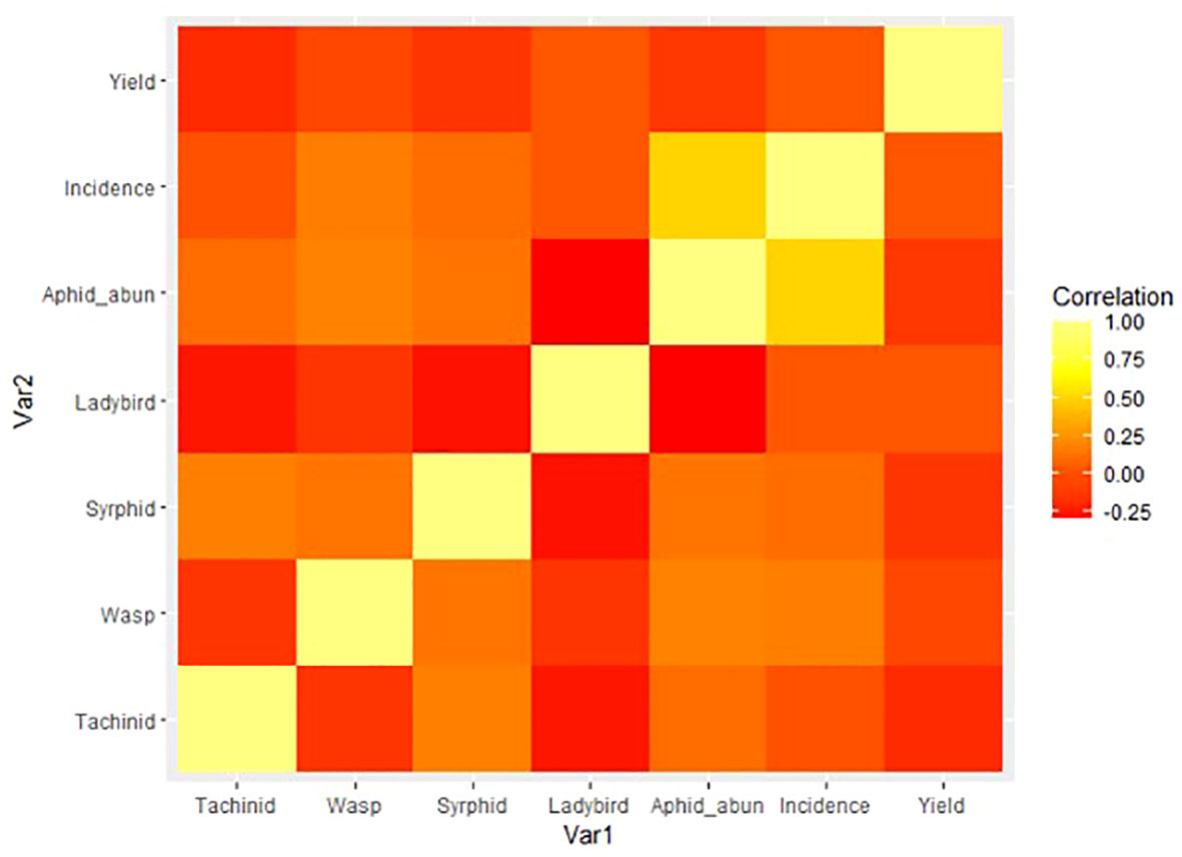
Heatmap of correlations between natural enemy, pest, and yield measures, where yellow = stronger relationship and red = no relationship.

## Discussion

This study showed that different genotypes of lablab vary in their susceptibility to aphid infestation and suggested that some scope for selecting genotypes for tolerance of aphid infestations is feasible. Aphid infestation was not linked to yield loss here although this is reported in lablab (3) and other legume crops (44). However, a greater diversity in lablab genotypes may provide growers with a wider range of traits including those for pest tolerance or resistance, which could reduce or slow the growth of the pest population (45). However, the presence or absence of FMV did not significantly influence pest incidence or populations of natural enemies of bean aphids on lablab. This finding was surprising but similar to that reported by Obanyi et al. (21) who also monitored natural enemy numbers associated with field margins comprising similar flowering species. Natural enemy numbers only increased by approximately 10% over field plots with no field margin. Karp et al. (46) reported that nearly half of natural enemies either are neutral or negatively respond to non-crop habitats and suggested that actual response enemies are variable spatially and by system and that more context is needed before habitat conservation can be expected to influence pest numbers.

In contrast, Woltz et al. (47) reported that the floral composition of landscapes and habitat management significantly influenced insect natural enemy numbers although this was on a different crop (soya bean). However, no interaction was noted between landscape context and local habitat management on the abundance of coccinellids. The findings from other studies have suggested that the degree to which the manipulation of landscapes can influence natural enemy communities and populations is highly dependent on the degree of heterogeneity in the surrounding landscape. Manipulation of margins was more effective for increasing richness and abundance in simplified, crop-dominated landscapes than in diverse ones for a variety of arthropods [e.g., pollinators generally (48), such as bees (49, 50), butterflies (51), and aphidophagous syrphids (52)]. Other studies have shown that taxon diversity and abundance respond primarily to landscape composition alone with habitat manipulation or management having a negligible influence. These contrasting results may indicate that these relationships are species- and context-dependent.

In the first cropping season, there was a weak tendency of FMV to support coccinellids compared to the second season. These data are consistent with Woltz et al. (47) who found a rise in the abundance of coccinellids in soybean fields with the proportion of seminatural habitat in the surrounding landscape. Similar positive effects of seminatural habitat on natural enemies have been found for a variety of predatory and parasitic taxa (53).

In this study, more natural enemies were captured at the center of the plots than on the field margins. However, this does not concur with the findings of other studies such as those of Walton and Isaacs (54) who found a significantly higher abundance of both syrphid flies and predatory wasps in blueberry fields that were adjacent to native wildflower plantings than next to mown grass strips. It is often assumed that most natural enemies will be located in the margin and periodically move into the crop to provide services, but here, it appears that the crop was a more conducive habitat for the natural enemies, perhaps due to being less exposed and more sheltered, and the edges of the crop could get hot and dry. It is also possible that the crop itself provided a range of resources (pollen, nectar, prey) that supported the natural enemies well. These results agree with Mwani et al. (55) who reported that monocropped lablab had the highest population of natural enemies compared to intercropped lablab with maize, suggesting that lablab provided a range of food resources for the natural enemies. Tscharntke et al. (56) also reported that natural habitats and crops can provide a suitable environment for many pests and natural enemy species at several key life stages. Again, the wider landscape context (10 m × 10 m plots) may have a more profound influence on these findings than the immediate surroundings. Other studies have similarly demonstrated predators and parasitoids alike, benefiting from managed landscapes without clear effects on adjacent fields. For instance, a border of guinea grass did not affect the abundance of predators within maize plants or the density of spotted stem borer, *Chilo partellus*, despite attracting sufficient predators to the strip (57). Inconsistencies in these findings could be a result of differing natural enemies’ biology, foraging behavior, or landscape composition. Assumptions in conservation biological control are that flowering plants coupled by the abundance of non-crop field margin near a crop will positively enhance populations of natural enemies and that the natural enemies will move into the crop from the margins, devour, parasitize, or simply immobilize the pests, thereby improving pest management leading to reduced yield losses. In this study, FMV had no effect on bean aphid abundance and severity of damage, contrary to Labruyere et al. (58) who found out that crop type and management as well as proximity of grass margins affected the abundance and the nutritional state of three abundant seed-eating carabid species.

Plant species in an agroecosystem influence the abundance of natural enemies, and in this study, lablab genotypes did not vary in their potential to support natural enemies. Important plant traits that greatly influence the abundance of natural enemies are the period of blooming, floral area, inflorescence size, hue, chroma, corolla size, and the number of open flowers (59). Studies should be oriented toward the exploration of different traits by plants for sustainable biological control in an agroecosystem through habitat management. Lablab genotypes significantly influenced aphid incidence, an indication that some genotypes could be resistant or less preferred by the aphids. Lablab genotypes influenced the number of pods, the number of peduncles, and peduncle length. However, there was no significant relationship between genotype and yield.

## Conclusion

This study showed that the presence of field margin vegetation was not associated with a higher number of natural enemies. However, we found no interaction of FMV with lablab genotypes, aphid abundance, and yield. However, as the studies reviewed above suggest, the influences of FMV on local habitats and natural enemy populations are likely system-specific and dependent upon the biology of the natural enemies under study. Bean aphids are not the only pests of lablab in the study sites, and other crops have additional pest-natural enemy associations that may be successfully managed at the local levels. A greater understanding of these complex relationships could enable farmers and researchers to develop more effective management options suited to specific landscapes, prevailing pests, and their natural enemy communities. The findings of this study highlight the important contribution of crop genotype together with the presence of field margin species in the regulation of aphids and their natural enemies in lablab bean. Thus, further research is required on the mechanisms of pest tolerance and the contribution of individual field margin species and life histories in the biological control of insect pests in tropical legumes.

## Data availability statement

The raw data supporting the conclusions of this article will be made available by the authors, without undue reservation.

## Author contributions

JK: Data curation, Investigation, Writing – original draft, Writing – review & editing, Formal analysis. JN: Methodology, Supervision, Writing – review & editing. RM: Methodology, Supervision, Writing – review & editing. JO: Conceptualization, Methodology, Supervision, Writing – review & editing. PB: Methodology, Project administration, Supervision, Writing – review & editing. EC: Methodology, Project administration, Supervision, Writing – review & editing. SA: Conceptualization, Formal analysis, Investigation, Methodology, Supervision, Writing – review & editing. SB: Conceptualization, Data curation, Formal analysis, Methodology, Supervision, Writing – review & editing. PS: Conceptualization, Data curation, Funding acquisition, Investigation, Supervision, Writing – original draft, Writing – review & editing.
